# Effect of *Streptococcus uberis* on Gamma Delta T Cell Phenotype in Bovine Mammary Gland

**DOI:** 10.3390/ani11123594

**Published:** 2021-12-19

**Authors:** Petr Slama, Terezie Zavadilova, Ales Pavlik, Pavel Horky, Sylvie Skalickova, Jiri Skladanka, Shubhadeep Roychoudhury, Simona Baldovska, Adriana Kolesarova, Roman Konecny, Vladimir Tancin, Monika Zouharova

**Affiliations:** 1 Department of Animal Morphology, Physiology and Genetics, Faculty of AgriSciences, Mendel University in Brno, Zemedelska 1, 613 00 Brno, Czech Republic; terezie.zavadilova@mendelu.cz (T.Z.); ales.pavlik@mendelu.cz (A.P.); 2 Department of Animal Nutrition and Forage Production, Faculty of AgriSciences, Mendel University in Brno, Zemedelska 1, 613 00 Brno, Czech Republic; pavel.horky@mendelu.cz (P.H.); sylvie.skalickova@mendelu.cz (S.S.); jiri.skladanka@mendelu.cz (J.S.); 3 Department of Life Science and Bioinformatics, Assam University, Silchar 788 011, India; shubhadeep1@gmail.com; 4 AgroBioTech Research Centre, Slovak University of Agriculture in Nitra, 94976 Nitra, Slovakia; simona.baldovska@uniag.sk; 5 Faculty of Biotechnology and Food Sciences, Institute of Applied Biology, Slovak University of Agriculture in Nitra, Tr. A. Hlinku 2, 94976 Nitra, Slovakia; adriana.kolesarova@uniag.sk; 6 Department of Animal Husbandry Sciences, Faculty of Agriculture, University of South Bohemia, Studentska 1668, 370 05 Ceske Budejovice, Czech Republic; konecnyroman@centrum.cz; 7 Faculty of Agrobiology and Food Resources, Institute of Animal Husbandry, Slovak Agriculture University in Nitra, Trieda Andreja Hlinku 2, 94976 Nitra, Slovakia; vladimir.tancin@nppc.sk; 8 NPPC—Research Institute for Animal Production, Hlohovecka 2, 95141 Luzianky, Slovakia; 9 Infectious Diseases and Preventive Medicine Department, Veterinary Research Institute, Hudcova 296/70, 621 00 Brno, Czech Republic; zouharova.m@vri.cz

**Keywords:** *Streptococcus uberis*, mastitis, apoptosis, lymphocyte, mammary gland, T cell

## Abstract

**Simple Summary:**

Bovine mastitis (inflammation of the mammary gland) is still an important problem for dairy farmers. This disease causes great financial losses across the world. The common method of treating mastitis is through the use of antibiotics. Antibiotic treatment should be minimized because of increasing antibiotic resistance. *Streptococcus uberis* (*S. uberis*) is one of the most important pathogens that causes bovine mastitis. This bacterium is able to hide and survive inside of epithelial cells. In this situation, antibiotic treatment is not efficient. Therefore, it is necessary to study the pathogenesis of mastitis that is caused by *S. uberis* to better understand how to treat this disease. In this study, we investigated a special type of lymphocytes—γδ T cells. The results of our study show that those cells may play a role in terminating inflammation in the mammary glands of cattle.

**Abstract:**

In this study, we focused analyzing γδ T cells during bovine mammary gland inflammation induced by *Streptococcus uberis*. A mammary gland cell suspension was obtained using lavage 24, 48, 72, and 168 h after intramammary-induced infection. The proportion of lymphocytes increased during the entire week in which inflammation was present. The γδ T cells were also elevated during inflammation, reaching their peak at 72 h following induced inflammation. The percentage of apoptotic lymphocytes continually increased, with the highest proportion occurring 168 h after *S. uberis* infection. The results show that γδ T cells may be involved in the resolution of inflammation in bovine mammary glands, with the apoptosis of those cells potentially playing an important role.

## 1. Introduction

*Streptococcus uberis* is one of the main pathogens that causes inflammation of the bovine mammary gland [1]. This bacterium has a very impressive mechanism for avoiding any contact with immune cells, mostly with phagocytes. It internalizes itself into the mammary epithelial cells, where it persists and hides, resulting in immune cells being unable to eliminate it [2]. For the internalization process, the pathogen uses the *Streptococcus uberis* adhesion molecule (SUAM) [3,4,5,6,7]. It is common knowledge that *S. uberis* is capable of affecting the viability of immune cells during the immune response of the bovine mammary gland. Neutrophil apoptosis is delayed, and subsequent efferocytosis by macrophages is also delayed by *S. uberis*. This can result in acute mammary gland inflammation transitioning to become chronic [8]. The same bacteria influence the apoptosis of lymphocytes. In our previous study, we investigated the apoptosis of lymphocytes, which can be delayed during experimentally induced mastitis by *S. uberis* [9] or in in vitro through the use of the lipopolysaccharide of *Escherichia coli* [10]. Contrary to that, the peptidoglycan of *Staphylococcus aureus* is able to induce lymphocyte apoptosis during the initial stages of mastitis [11]. Similar results have been obtained in experiments involving the lipopolysaccharide of *E. coli* or muramyl dipeptide [12].

In our previous studies [9,11,12], we have only investigated whole lymphocyte populations. Each s lymphocyte subpopulation may have a different sensitivity level for the induction of apoptosis. In humans, gamma delta T (γδ T) cells represent a small lymphocyte population. In cattle, there is a higher proportion of these cells compared to the proportion that is human lymphocyte populations [13,14]. *S. aureus* is able to change the proportion of γδ T cells during the inflammatory response of the bovine mammary gland [15]. The increase in the proportion of that lymphocyte subpopulation is also correlated with the increased lymphocyte apoptosis [16].

γδ T lymphocytes are a group of T cells that have γ and δ chains on their surface. These cells represent 0.5 to 5% of all human T cells [14]. Contrary to that, γδ T cells make up 15 to 60% of all of the circulating lymphocytes in cattle [13]. Bovine models can also be used to clarify the role of γδ T cells in infectious diseases in humans [17,18]. γδ T cells have various functions, such as the cytokine production, antigen presentation, and immune response regulation. Cattle γδ T cells are also involved in immune suppression [13]. In the intact bovine mammary gland, it has been found that CD2-positive γδ T (CD = cluster of differentiation) cells are more dominant than CD2-negative cells. Despite previous investigations, the exact role of γδ T cells in the pathogenesis of bovine mammary gland inflammation is not known [19].

The goal of this study was to evaluate the effect of *S. uberis* on the γδ T cell phenotype in lymphocytes during the inflammatory response of the bovine mammary gland.

## 2. Materials and Methods

### 2.1. Animals and Experimental Design

For our experiments, we used 10 crossbred virgin heifers that were about 18 months old: a total of five heifers were used for the experiment with *S. uberis,* and five heifers were used as the control group. The animal experiments were approved by the Branch Commission for Animal Welfare of the Ministry of Education, Youth and Sports of the Czech Republic (MSMT-11516/2019-2). The experimental animals were free of intramammary infections, which was demonstrated by means of a bacteriological examination. This examination was performed through a culture of lavages on blood agar (5% washed ram erythrocytes) with aerobic incubation for 24 h (37 °C). For the control group, we used phosphate-buffered saline (PBS; Sigma, Saint Louis, MO, USA) at a volume of 20 mL for each mammary gland in the udder. The mammary gland sinuses were washed out with PBS to obtain a cell suspension, that was similar to the one that was used in a previously reported upon procedure [8,9]. In short, the first sample was obtained by a lavage of one quarter after 24 h following instillation with PBS, and the remaining quarters were washed out at 48 h, 72 h, and 168 h following the use of PBS [9,12]. The percentage of lymphocytes obtained from the lavages was analyzed by flow cytometry in dot plots with forward scatter and side scatter [20].

### 2.2. Induction of Inflammatory Response

In our experiments, we used *S. uberis* that had been isolated from a subclinical case of mastitis (CCM 4617; Czech Collection of Microorganisms, Masaryk University, Brno). An amount of 1 mL of the *S. uberis* stock culture was inoculated onto a cellophane membrane, which was placed on a ram blood agar and incubated for 4 h to obtain bacteria in the exponential growth phase. After that, the bacterial suspensions were harvested, washed once with PBS, and adjusted to a final concentration that was equal to 8 × 10^6^ CFU (colony forming unit)/mL PBS. After the bacterial suspensions were diluted to to 800 CFU/mL, the inocula were adjusted into the syringes to be used for instillation into the mammary glands. Each mammary quarter was injected with 5 mL of inoculum (800 CFU/mL) through the teat orifice using a syringe and a catheter (AC5306CH06, Porges S.A., Le Plessis Robinson, France). A few days after innoculation, samples were collected as previously described (in Section 2.1) by lavages using 20 mL of PBS.

### 2.3. Flow Cytometry Analysis of Apoptosis and γδ T Lymphocytes

Apoptotic lymphocytes were analyzed by flow cytometry (BriCyte E6, Mindray, Shenzhen, China) following staining with Annexin-V (FITC) and propidium iodide (PI) [21] using the Annexin-V-FLUOS staining kit (Boehringer Mannheim, GmbH, Mannheim, Germany). The cell suspensions were analyzed by flow cytometry with differentiation of 20,000 cells. The lymphocytes were distributed over three different quadrants of the dot plots, representing viable (Annexin-V−/PI−), apoptotic (Annexin-V+/PI−), and necrotic lymphocytes (Annexin-V+/PI+). The dot plots were assessed using MR Flow software (Mindray, China).

For the analysis of the γδ T cells, mouse monoclonal anti-bovine γδ TCR (T cell receptor) (GB21A, IgG2b; Serotec Ltd., Oxford, UK) antibody was used [19,22]. All of the mAbs (monoclonal antibodies) were from Serotec Ltd., Oxford, UK. FITC-conjugated goat anti-mouse IgG2b (Southern Biotechnology Associated, Inc., Birmingham, AL, USA) was used as the secondary antibody [19].

### 2.4. Statistical Analysis

Arithmetic means and standard deviations were used to describe the percentage of lymphocytes, apoptotic lymphocytes, and γδ T lymphocytes. Statistically significant differences in the portions of the mentioned parameters were assessed using the paired t-test. The relationship between lymphocyte and γδ T lymphocyte apoptosis was ascertained by correlation analysis. The data were analyzed using STATISTICA 8.1 software (StatSoft CR Ltd., Prague, Czech Republic).

## 3. Results

### 3.1. The Proportion of Lymphocytes during Inflammatory Response

**Figure 1:**
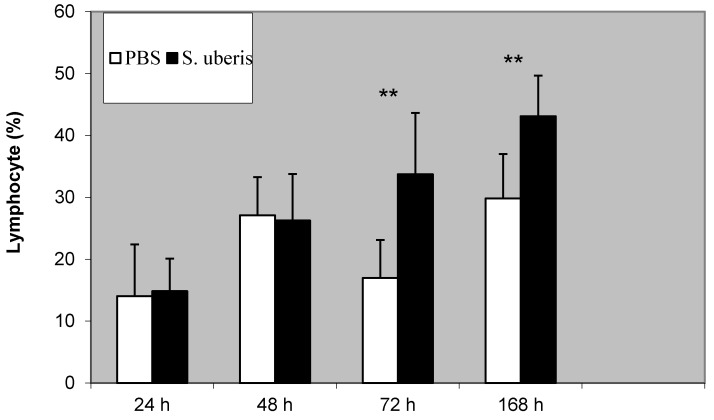
The proportion of lymphocytes following induction of bovine mammary glands with PBS (control) and *S. uberis*. Statistically significant differences between control (PBS) and *S. uberis* are marked with asterisks (** *p* < 0.01).

The proportion of lymphocytes increased during the inflammatory response. We noted significant differences between the control group and in the group that with *S. uberis*-induced inflammation at 72 and 168 h following induction, respectively (*p* < 0.01) (Figure 1).

### 3.2. The Proportion of γδ T Cells during Inflammatory Response

**Figure 2:**
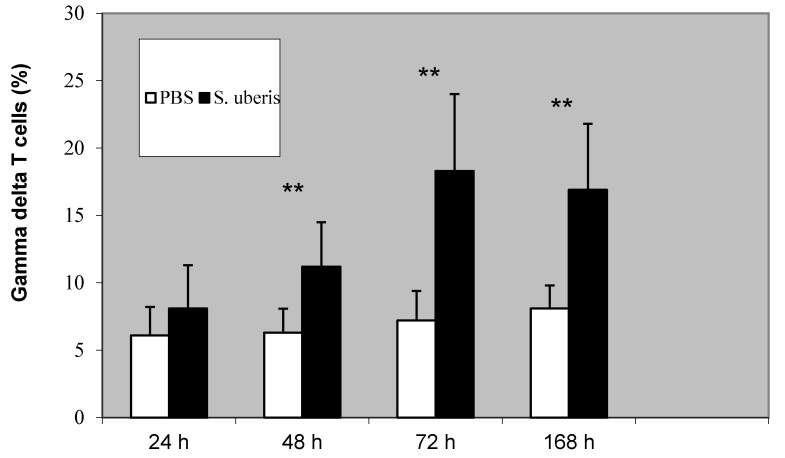
The proportion of γδ T cells following the induction of bovine mammary gland with PBS (control) and *S. uberis*. Statistically significant differences between the control (PBS) and *S. uberis* are marked with asterisks (** *p* < 0.01).

The proportion of γδ T cells changed *S. uberis* was used to induce inflammation compared to when PBS treatment was administered. After the use of PBS, the percentage of γδ T cells was similar at all time points. After the *S. uberis* induction of the inflammatory response, the proportion of γδ T cells was significantly higher at 48, 72, and 168 h (*p* < 0.01) (Figure 2).

### 3.3. The Proportion of Lymphocyte Apoptosis during Inflammatory Response

**Figure 3:**
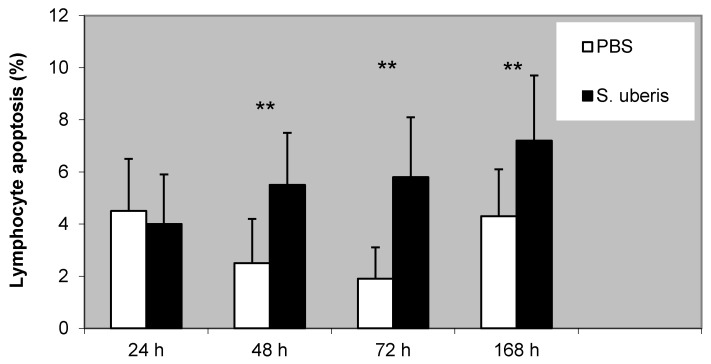
The proportion of apoptotic lymphocytes following the induction of bovine mammary glands with PBS (control) and *S. uberis*. Statistically significant differences between control (PBS) and *S. uberis* are marked with asterisks (** *p* < 0.01).

Lymphocyte apoptosis was gradually increased during the inflammatory response caused by *S. uberis*. We found a significantly higher proportion of apoptotic lymphocytes after the administration of *S. uberis* to the mammary gland in comparison to PBS treatment (*p* < 0.01) (Figure 3).

We also noted a high correlation between lymphocyte apoptosis and the proportion of γδ T cells that were observed after induction using *S. uberis* (r^2^ = 0.870; *p* < 0.01).

## 4. Discussion

In the present study, we analyzed the effect of *S. uberis* on the proportion of γδ T cells during the experimentally induced inflammatory response of the bovine mammary gland. We also detected the proportion of lymphocytes in the differential cell count and the proportion of apoptotic lymphocytes from mammary lavages following the intramammary instillation of *S. uberis*.

The lymphocyte portion gradually increased following *S. uberis* instillation into the mammary glands compared to in the controls (instillation of PBS). A similar result was noted in a previous study using *S. aureus*, *S. uberis*, lipopolysaccharides, and muramyl dipeptide to induce an inflammatory response in bovine mammary glands [9,12,23].

We investigated changes in the presence of γδ T cells during the inflammation of the bovine mammary gland that had been experimentally induced by *S. uberis*. We found that the proportion of γδ T cells was gradually increased during the inflammatory response and that this was highly correlated with the increase that was observed in the apoptotic lymphocytes. Previously, we noted that *S. aureus* had a similar effect on the γδ T-cell population [15], with similarities being correlated with an increase in apoptotic cells [12]. Faldyna et al. [19] stated that there is a lack of information about the role of γδ T cells during mammary gland inflammation. They also mentioned that it seems that γδ T cells may be involved in the first phase of the mammary gland’s inflammatory response. In contrast to that, we determined that the percentage of γδ T cells increased at 72 and 168 h following the experimental infection of the bovine mammary glands by *S. uberis*. It seems that γδ T cells could have any function in the resolution of the inflammation of the bovine mammary gland.

It is well known that dendritic cells are able to present antigens to T cells [24]. On the other hand, γδ T cells are able to induce the maturation of dendritic cells as well as their production of cytokines and the expression of the CD receptors on their surface [25]. When macrophages are less efficient during the phagocytosis of apoptotic cells (efferocytosis), there is increased clearing of apoptotic cells by dendritic cells [26]. In our opinion, this mechanism could contribute to the effective resolution of inflammation. Crosstalk between dendritic cells and γδ T cells may be included in that process. Above, we have noted that γδ T cells can induce the production of cytokines by dendritic cells, but it was also shown that dendritic cells induce cytokine production by γδ T cells [27]. Engulfing apoptotic cells with dendritic cells may cause a higher production of anti-inflammatory cytokines, such as IL-10 and TGF-β [28]. Higher TGF-β1 production was previously found between 40 and 72 h after experimentally induced mammary gland inflammation with *S. aureus* [29]. An *S. aureus*-induced chronic mammary gland infection also modifies the expression of the TGF-β subfamily components during active involution [30]. IL-10 was also detected at a high concentration between 24 and 48 h after *E. coli* was used to experimentally induce mammary gland inflammation [31]. Altogether, it seems that increasing the proportion of γδ T cells and apoptotic lymphocytes may contribute to the resolution of inflammation in the bovine mammary gland. The results of the authors Espinosa-Cueto et al. [28] show another example of the crosstalk between dendritic cells and γδ T cells. They determined that dendritic cells phagocytosing apoptotic macrophages activate CD8-positive T cells [28]. The activation of γδ T cells is also promoted by the necroptosis of dendritic cells [32]. Necroptosis is a regulated form of necrosis that has an important role in inflammation [33].

## 5. Conclusions

In summary, we hypothesize that the gradual increase in γδ T cells seen during the inflammatory response of the bovine mammary gland could be connected to the transition of innate to adaptive immunity. These cells could interact with dendritic cells, and this interaction could act as the bridge between innate and adaptive immunity. Follow-up explorations must be conducted to answer the many remaining questions that are related to the interaction between γδ T cells and dendritic cells during the inflammatory response of the bovine mammary gland.

## Data Availability

All relevant data are within the manuscript.
